# Maternal near miss and mortality in a rural referral hospital in northern Tanzania: a cross-sectional study

**DOI:** 10.1186/1471-2393-13-141

**Published:** 2013-07-04

**Authors:** Ellen JT Nelissen, Estomih Mduma, Hege L Ersdal, Bjørg Evjen-Olsen, Jos JM van Roosmalen, Jelle Stekelenburg

**Affiliations:** 1 Haydom Lutheran Hospital, 9000 Haydom, Manyara, Tanzania; 2 Athena Institute, Faculty of Earth and Life Sciences, VU University Amsterdam, de Boelelaan 1085, 1081 HV Amsterdam, Netherlands; 3 Stavanger Acute Medicine Foundation for Education and Research (SAFER), Stavanger University Hospital, POB 8100, 4068 Stavanger, Norway; 4 Centre for International Health, University of Bergen, Årstadveien 21, N-5009 Bergen, Norway; 5 Department of Obstetrics and Gynaecology, Sørlandet Hospital, Engvald Hansens vei 6, 4400 Flekkefjord, Norway; 6 Department of Obstetrics, Leiden University Medical Centre, Albinusdreef 2, 2333 Leiden, ZA, Netherlands; 7 Department of Medical Humanities, EMGO Institute for Health and Care Research, VU University Medical Centre, Van der Boechorststraat 7, 1081 BT Amsterdam, Netherlands; 8 Department of Obstetrics & Gynaecology, Leeuwarden Medical Centre, Henri Dunantweg 2, 8934 AD Leeuwarden, Netherlands

**Keywords:** Maternal near miss, Severe acute maternal morbidity, Maternal mortality, Quality of obstetric care, WHO near miss approach, WHO near miss criteria

## Abstract

**Background:**

Maternal morbidity and mortality in sub-Saharan Africa remains high despite global efforts to reduce it. In order to lower maternal morbidity and mortality in the immediate term, reduction of delay in the provision of quality obstetric care is of prime importance. The aim of this study is to assess the occurrence of severe maternal morbidity and mortality in a rural referral hospital in Tanzania as proposed by the WHO near miss approach and to assess implementation levels of key evidence-based interventions in women experiencing severe maternal morbidity and mortality.

**Methods:**

A prospective cross-sectional study was performed from November 2009 until November 2011 in a rural referral hospital in Tanzania. All maternal near misses and maternal deaths were included. As not all WHO near miss criteria were applicable, a modification was used to identify cases. Data were collected from medical records using a structured data abstraction form. Descriptive frequencies were calculated for demographic and clinical variables, outcome indicators, underlying causes, and process indicators.

**Results:**

In the two-year period there were 216 maternal near misses and 32 maternal deaths. The hospital-based maternal mortality ratio was 350 maternal deaths per 100,000 live births (95% CI 243–488). The maternal near miss incidence ratio was 23.6 per 1,000 live births, with an overall case fatality rate of 12.9%. Oxytocin for prevention of postpartum haemorrhage was used in 96 of 201 women and oxytocin for treatment of postpartum haemorrhage was used in 38 of 66 women. Furthermore, eclampsia was treated with magnesium sulphate in 87% of all cases. Seventy-four women underwent caesarean section, of which 25 women did not receive prophylactic antibiotics. Twenty-eight of 30 women who were admitted with sepsis received parenteral antibiotics. The majority of the cases with uterine rupture (62%) occurred in the hospital.

**Conclusion:**

Maternal morbidity and mortality remain challenging problems in a rural referral hospital in Tanzania. Key evidence-based interventions are not implemented in women with severe maternal morbidity and mortality. Progress can be made through up scaling the use of evidence-based interventions, such as the use of oxytocin for prevention and treatment of postpartum haemorrhage.

## Background

Despite global efforts through the Safe Motherhood Initiative and the declaration of the Millennium Development Goals (MDG), there is only a slight decline in maternal mortality in sub-Saharan Africa [1,2]. Although Tanzania shows progress towards improving maternal health, as is shown by a 47% decline in maternal mortality ratio (MMR) between 1990 and 2010 (from an estimated MMR of 870 per 100,000 live births in 1990 to a MMR of 460 per 100,000 live births in 2010), maternal mortality remains high [3]. The aim of MDG 5 is to reduce maternal mortality by 75% between 1990 and 2015 [4]. Although the numbers of maternal deaths (MD) around the globe are high, absolute numbers in hospitals are low. Therefore, including cases of women who almost die, but survive pregnancy-related complications, is progressively being used to study the quality of obstetric care [5-8]. These near miss cases represent most of the characteristics of maternal deaths [9]. Combined, they create larger numbers and may lead to more rapid and precise reporting. Since identification criteria for near miss cases are not uniform and studies thus not comparable [6,7], WHO developed a new definition of maternal near miss (MNM) and identification criteria for maternal near miss cases in 2009 [10]. This resulted in the “WHO near miss approach for maternal health” in 2011 [11].

Currently, in Tanzania half of the deliveries take place in health facilities and 51% of all deliveries are assisted by a skilled provider [12]. Unfortunately skilled birth attendance does not necessarily equal good quality of care [13]. Many evidence-based effective approaches are available to prevent and treat obstetric emergencies, but their implementation lags behind [14,15]. Human resources are scarce and policymakers encourage scaling up facility deliveries. Increased utilization of skilled birth attendants at facilities with insufficient human resources may further threaten quality of care. Negative perception of the quality of care by the community inhibits the decision to deliver in a facility [16,17] and so a vicious circle is created. When scaling up skilled birth attendance, there is a need for a well-functioning health system with sufficient resources and a good infrastructure. Both, quantity of human resources and quality of care should be addressed [13,18].

In order to reduce maternal mortality and severe acute maternal morbidity, it is imperative not only to increase the numbers of skilled personnel, but also to improve access to health institutions and to reduce the delay in providing quality obstetric care within institutions. Quality of obstetric care can be measured using outcome indicators, such as case fatality rate (CFR), and process indicators, such as the use of oxytocin after delivery to prevent postpartum haemorrhage [11,19].

This study aims to assess the occurrence of severe maternal morbidity and mortality in a referral hospital in rural Tanzania as proposed by the WHO near-miss approach and to assess implementation levels of key evidence-based interventions in women experiencing severe maternal morbidity and mortality.

## Methods

This study is part of an intervention study. Data that were collected serve as baseline for an intervention with simulation-based training in routine delivery care, prevention and treatment of postpartum haemorrhage (PPH). This was a prospective cross-sectional study, conducted from November 2009 until November 2011 in Haydom Lutheran Hospital (HLH), a referral hospital in rural Northern Tanzania. HLH is a 400-bed hospital owned by the Mbulu Diocese of the Evangelical Lutheran Church in Tanzania. The hospital provides free reproductive and child health services, comprehensive emergency obstetric care, including ambulance and radio service. Furthermore there is an Intensive Care Unit (ICU) with 24-hours medical supervision and mechanical ventilation. Annually there are around 5000 deliveries. Extrapolating from the 2002 census, the immediate catchment area was covering a population of approximately 327,000 in 2010 [20]. The greater reference area covered a population of approximately 2,200,000 people [20].

### Outcome measures

The primary outcome measures were the total number of MD, MNM and live births (LB) in the hospital during the study period. Subsequently, outcome indicators were calculated such as the number of women with life-threatening conditions. As proposed by the WHO approach, overall near miss indicators were calculated such as the severe maternal outcome ratio, the maternal near miss incidence ratio, the maternal near-miss mortality ratio, and the case fatality rate. Hospital access indicators were calculated such as the number of women with life-threatening conditions at arrival, the proportion of these women among all women with life-threatening conditions, the proportion of these women coming from other hospitals, and the women with life-threatening conditions at arrival mortality index. Lastly, intra hospital care indicators were calculated: the number of maternal near misses and deaths who developed these conditions in the hospital and the intra hospital mortality index.

Implementation levels of evidence-based interventions were measured, such as use of oxytocin for prevention and treatment of PPH, use of magnesium sulphate for treatment of eclampsia, use of prophylactic antibiotics during caesarean section, use of parenteral antibiotics for treatment of sepsis, and the proportion of women with uterine rupture that occurred in the hospital.

### Inclusion criteria

All maternal deaths and maternal near misses that were admitted to HLH were prospectively included in the study during the above-mentioned period. A maternal death is defined as: the death of a woman while pregnant or within 42 days of termination of pregnancy from any cause. A maternal near miss is defined as: a woman who nearly died but survived a complication that occurred during pregnancy, childbirth or within 42 days of termination of pregnancy [10]. The intention was to use the WHO near miss criteria for the identification of maternal near misses. However, not all WHO criteria were applicable, and the identification criteria were adapted to the local situation in HLH as is described elsewhere [21]. Table 1 shows an overview of the WHO near miss criteria and the Haydom modification.

**Table 1:** **WHO near miss criteria adapted to the local context of HLH (reproduced from Nelissen et al.**)

**WHO near miss criteria**	**Haydom near miss criteria**
**Clinical criteria**	
Acute cyanosis	Acute cyanosis
Gasping	Gasping
Respiratory rate > 40 or < 6/min	Respiratory rate > 40 or < 6/min
Shock	Shock ^a^
Oliguria non responsive to fluids or diuretics	Oliguria non responsive to fluids or diuretics ^b^
Failure to form clots	Failure to form clots ^c^
Loss of consciousness lasting > 12 h	Loss of consciousness lasting > 12 h ^d^
Cardiac arrest	Cardiac arrest ^e^
Stroke	Stroke ^f^
Uncontrollable fit/total paralysis	Uncontrollable fit/total paralysis ^g^
Jaundice in the presence of pre-eclampsia	Jaundice in the presence of pre-eclampsia ^h^
**Laboratory-based criteria**	
Oxygen saturation < 90% for ≥ 60 minutes	Oxygen saturation < 90% for ≥ 60 minutes
PaO2/FiO2 < 200 mmHg	
Creatinine ≥ 300 μmol/l or ≥ 3.5 mg/dL	
Bilirubin > 100 μmol/l or > 6.0 mg/dL	
pH < 7.1	
Lactate > 5 mEq/mL	
Acute thrombocytopenia (< 50,000 platelets/ml)	Acute thrombocytopenia (< 50,000 platelets/ml)
Loss of consciousness and ketoacids in urine	
**Management-based criteria**	
	Admission to intensive care unit
Use of continuous vasoactive drugs	
Hysterectomy following infection or haemorrhage	Hysterectomy following infection or haemorrhage
Transfusion of ≥ 5 units of blood	Transfusion of ≥ 1 unit of blood
Intubation and ventilation for ≥ 60 minutes not related to anaesthesia	Intubation and ventilation for ≥ 60 minutes not related to anaesthesia
Dialysis for acute renal failure	
Cardio-pulmonary resuscitation	Cardio-pulmonary resuscitation
**Severe maternal complications**	
	Eclampsia ^i^
	Sepsis or severe systemic infection ^j^
	Uterine rupture ^k^

Applicability of the WHO near miss criteria in a low resource setting. PLoS One 2013, 8:e61248.

a: Shock is defined as a persistent severe hypotension, defined as a systolic blood pressure < 90 mmHg for 60 min with a pulse rate of ≥ 120/min despite aggressive fluid replacement (> 2 L).

b: Oliguria is defined as an urinary output < 30 ml/hour for 4 hours or < 400 ml/24 hr.

c: Failure to form clots is defined as the absence of clotting from the IV site after 7–10 minutes.

d: Unconsciousness/coma lasting > 12 hours is defined as a profound alteration of mental state that involves complete or near-complete lack of responsiveness to external stimuli or Glasgow Coma Scale < 10.

e: Cardiac arrest is defined as loss of consciousness and absence of pulse or heart beat.

f: Stroke is defined as a neurological deficit of cerebrovascular cause that persists ≥ 24 hours, or is interrupted by death within 24 hours.

g: Uncontrollable fit is a condition in which the brain is in state of continuous seizure.

h: Pre-eclampsia: the presence of hypertension associated with proteinuria. Hypertension is defined as a blood pressure ≥ 140 mmHg (systolic) or ≥ 90 mmHg (diastolic). Proteinuria is defined as excretion of ≥ 300 mg protein/24 hr or 300 mg protein/litre urine or ≥ 1+ on a dipstick.

i: Eclampsia is defined as the presence of hypertension associated with proteinuria and fits. Hypertension is defined as a blood pressure ≥ 140 mmHg (systolic) or ≥ 90 mmHg (diastolic). Proteinuria is defined as excretion of ≥ 300 mg protein/24 hr or 300 mg protein/litre urine or ≥ 1+ on a dipstick.

j: Sepsis is defined as a clinical sign of infection and 3 of the following: temp > 38°C or < 36°C, respiration rate > 20/min, pulse rate > 90/min, WBC >12.

k: Uterine rupture is defined as the complete rupture of a uterus (including peritoneum) with (partial) extrusion of the fetus during labour.

### Data collection

Cases were identified on a daily basis by either the principal investigator (EN) or by one of the two trained research assistants (nurse-midwives). This was achieved through daily participation in the morning report and daily visits to the maternity ward, ICU and the internal medicine ward. When the inclusion criteria were met, a structured data abstraction form was filled out by the principal investigator or a research assistant. Data were obtained from the patient record. The facility medical staff was questioned in case of doubt or missing information. General information and obstetric details were collected. For the hospital access and intra hospital care indicators, the following information was registered: health status on arrival, maternal death within 24 hours, and referral status. For each case, one underlying cause was identified that started the cascade that led to maternal morbidity or mortality [22]. For example, a primipara was admitted with obstructed labour and had a caesarean section. After caesarean section she developed sepsis. The underlying cause that started the cascade that led to maternal morbidity or mortality was obstructed labour. This table shows one diagnosis per woman (mutually exclusive, totally inclusive). For the process indicators, information on preventive measures (measuring of vital signs, use of oxytocin or other uterotonics for prevention of PPH, use of prophylactic antibiotics during caesarean section) were registered, as well as the use of interventions (use of oxytocin or other uterotonics as treatment for PPH, IV-infusion, blood products, hysterectomy, magnesium sulphate or other anticonvulsant in case of eclampsia, parenteral therapeutic antibiotics, and laparotomy for uterine rupture).

### Quality assessment of the data

The completed data abstraction forms were checked by a second person on missing data or discrepancies. If needed, a copy of the hospital file was checked to validate the recordings. All MD cases were reviewed by a selection of the authors (EN, BEO, JVR and JS), as well as a random selection of the MNM cases and those cases, which were difficult to classify into an underlying cause. Observation bias was addressed by means of auditing all MD cases and a random sample of the MNM cases by four authors (EN, BEO, JVR, JS) until consensus was reached. All data were double entered and cross-checked in Epidata [23].

### Statistical analysis

Data analysis consisted of frequencies of demographic and clinical variables and underlying causes. Demographic variables were cross-tabulated for maternal outcome at discharge, and compared using chi-square test for categorical variables and t-test for continuous variables. Outcome indicators were calculated using the total number of live births during the study period and the total number of MNM and MD in that same period. Descriptive frequencies were calculated for underlying causes and process indicators. All results are reported as number (n) and frequency (%). Analysis was performed using SPSS Statistics, version 20 (SPSS Inc. Chicago, Illinois).

### Ethical approval

Ethical approval was obtained from the Tanzanian National Institute for Medical Research (NIMR) (reference NIMR/HQ/R.8a/Vol.IX/1247), the Tanzania Commission for Science and Technology (COSTECH) (reference 2012-56-NA-2011-201), and from the VU university medical center (VUmc), the Netherlands (reference 2011/389). Data were collected and extracted from patient records without identification of the subject. Data abstraction forms were filled in after discharge or death and therefore study inclusion did not have effect on the treatment. Considering these precautions, individually obtained informed consent was not required.

## Results

In the two-year study period 248 women with life-threatening conditions were included at Haydom Lutheran Hospital: 216 maternal near miss cases and 32 maternal deaths. In the study period 9471 deliveries and 9136 live births occurred at HLH.

Demographic and clinical characteristics are shown in Table 2. There were no statistical differences in characteristics between the group of maternal near miss and the group of maternal deaths. The Datoga tribe, who are pastoralists, experienced relatively more deaths than near misses, but this did not reach statistical significance. Almost 20% of all near misses (n = 40) and 22% of all maternal deaths (n = 7) had a gestational age of less than 24 weeks on admission. For 42% of all near misses (n = 91) gestational age was 36 weeks or more, compared to 25% of all maternal deaths (n = 8). The most common mode of delivery was vaginal delivery: 37% MNM (n = 80) and 47% MD (n = 15). This was followed by caesarean section: 32% MNM (n = 69) and 16% MD (n = 5). There were no assisted vaginal deliveries (e.g. forceps/vacuum extraction). Almost 30% of all women who died (n = 9) died undelivered.

**Table 2:** Demographic and clinical characteristics

	**MNM**	**MD**	**p-value**
	**n = 216**	**n = 32**	
Age mean (SD) in years	28.1 (6.3)	29.4 (6.1)	p = 0.266*
< 20 years n (%)	20 (9%)	3 (9%)	
20-35 years n (%)	150 (69%)	20 (63%)	
≥ 35 years n (%)	46 (21%)	9 (28%)	
Marital status n (%)			p = 0.341***
Single/separated/divorced	21 (10%)	5 (16%)	
Married/cohabiting	188 (87%)	25 (78%)	
Unknown	7 (3%)	2 (6%)	
Tribe n (%)			p = 0.477**
Datoga	31 (14%)	7 (22%)	
Iraqw	114 (53%)	14 (44%)	
Other	61 (28%)	9 (28%)	
Unknown	10 (5%)	2 (6%)	
Parity n (%)			p = 0.742**
0	47 (22%)	5 (16%)	
1-2	52 (24%)	7 (22%)	
≥ 3	100 (46%)	16 (50%)	
Unknown	17 (8%)	4 (13%)	
Previous caesarean section n (%)			p = 0.307***
No	145 (67%)	24 (75%)	
Yes	41 (19%)	3 (9%)	
Unknown	30 (14%)	5 (16%)	
Gestational age n (%)			p = 0.106**
< 24 weeks	40 (19%)	7 (22%)	
24-36 weeks	40 (19%)	10 (31%)	
≥ 36 weeks	91 (42%)	8 (25%)	
Unknown	45 (21%)	7 (22%)	
Mode of delivery n (%)			****
Vaginal delivery	80 (37%)	15 (47%)	
Caesarean section	69 (32%)	5 (16%)	
Laparotomy for uterine rupture	16 (7%)	1 (3%)	
Abortion/curettage	22 (10%)	2 (6%)	
Laparotomy for ectopic pregnancy	19 (9%)	-	
Undelivered	9 (4%)	9 (28%)	
Unknown	1 (1%)	-	
Fetal presentation n (%)			****
Cephalic	110 (51%)	15 (47%)	
Breech	10 (5%)	1 (3%)	
Other	9 (4%)	1 (3%)	
Abortion	40 (19%)	4 (13%)	
Unknown	47 (22%)	11 (34%)	

* Independent sample t-test; ** Chi-square test; *** Fisher exact test (2-sided); **** p-value could not be calculated due to too few cases.

As is shown in Table 3, there were 27.1 MNM and MD per 1,000 live births; this is also referred to as the severe maternal outcome ratio. For every maternal death there were 6.8 near miss cases. Hospital-based MMR was 350 per 100,000 live births (95% CI 243–488), with an overall CFR of 12.9%. Nearly 70% of all women with life-threatening conditions were ill on arrival and 20.9% were referred from another hospital. Death within 24 hours occurred in 7.6% (n = 13) of the women that were ill on arrival. Seventy-six women (30.6%) developed a life-threatening condition in the hospital, and four (5.3%) of these women died as a result of this condition.

**Table 3:** Outcome indicators

**Outcomes**	**Near-miss indicators**
All live births in the hospital (n)	9136
**Severe maternal outcomes**	
Maternal deaths (n)	32
Maternal near miss cases (n)	216
Women with life-threatening conditions (WLTC) (n)	248
**Overall near miss indicators**	
Severe maternal outcome ratio (SMOR) (per 1,000 live births)	27.1
Maternal near miss incidence ratio (per 1,000 live births)	23.6
Maternal near miss mortality ratio	6.75
Case fatality rate (%)	12.9%
**Hospital access indicators**	
WLTC at hospital arrival (n)	172
Proportion WLTC at arrival among all WLTC (%)	69.4%
Proportion of WLTC at arrival coming from other hospitals (%)	20.9%
WLTC at arrival mortality index (%)	7.6%
**Intra hospital care indicators**	
Intra hospital WLTC cases (n)	76
Intra hospital WLTC rate (per 1,000 live births)	8.32
Intra hospital mortality index (%)	5.3%

Live birth (LB): the complete expulsion or extraction from its mother of a product of conception, irrespective of the duration of the pregnancy, which, after such separation, breathes or shows any other evidence of life. Each product of such a birth is considered live born.

Women with life-threatening conditions (WLTC): the sum of maternal near miss and maternal deaths. (WLTC = MNM + MD).

Severe Maternal Outcome Ratio (SMOR): the number of women with life‒threatening conditions per 1,000 live births. [SMOR = (MNM + MD)/LB].

MNM incidence ratio: the number of maternal near miss cases per 1,000 live births. [MNM IR = MNM/LB].

Maternal near miss : mortality ratio: the proportion between maternal near miss cases and maternal deaths. [MNM : 1 MD].

Case fatality Rate: the number of maternal deaths divided by the number of women with life-threatening conditions, expressed as a percentage. [CFR = MD/(MNM + MD)].

WLTC at hospital arrival: the number of women with life‒threatening conditions who are ill on arrival.

Proportion WLTC at arrival among all WLTC: the number of WLTC who are ill on arrival divided by the total number of WLTC.

Proportion of WLTC at arrival coming from other hospitals: the number of WLTC who are ill on arrival and coming from another hospital divided by the total number of WLTC at arrival.

WLTC at arrival mortality index: the maternal deaths within 24 hours after arrival (MD24) divided by the number of women with life-threatening conditions who were ill on arrival, expressed as percentage. [WLTC at arrival MI = MD24/(WLTC at arrival)].

Intra hospital WLTC cases: the number of women with life-threatening conditions who developed these life-threatening conditions in the hospital.

Intra hospital WLTC rate (per 1000 live births): the number of women with life-threatening conditions who developed these life-threatening conditions in the hospital per 1000 live births.

Intra hospital mortality index: the number of maternal deaths who were not ill on arrival, divided by the number of women with life-threatening conditions who were not ill on arrival, expressed as percentage.

Table 4 demonstrates the underlying causes for both groups (MNM and MD) and the associated CFR per underlying cause. The major cause of morbidity and mortality is postpartum haemorrhage, accounting for 27% of all underlying causes, followed by abortion related complications (17%), obstructed labour (12%), ante partum haemorrhage (11%), and hypertensive disorders (9%). Women who got severely ill from an unknown cause had the highest risk of dying with a CFR of 80%, followed by cancer (67%) and non-obstetric infectious diseases (63%).

**Table 4:** Underlying causes of maternal morbidity and mortality (mutually exclusive, totally inclusive)

		**n (%)**	CFR (%)*
Indirect	Anaemia in pregnancy	19 (8%)	0%
	Infectious disease	11 (4%)	63%
	Cardiac disease	4 (2%)	50%
	Cerebral disease	2 (1%)	50%
Direct	Postpartum haemorrhage	67 (27%)	9%
	Abortion related complication	43 (17%)	7%
	Ante partum haemorrhage	26 (11%)	8%
	Hypertensive disorders	21 (9%)	10%
	Obstructed labour with uterine rupture	15 (6%)	13%
	Obstructed labour without uterine rupture	15 (6%)	7%
	Uterine scar rupture without obstructed labour	6 (2%)	0%
	Puerperal sepsis	9 (4%)	0%
	Anaesthesia related complication	1 (0.4%)	0%
Co-incidental	Cancer	3 (1%)	67%
	Trauma	1 (0.4%)	0%
Unknown	Unknown/undetermined cause	5 (2%)	80%

* *CFR:* case fatality rate, calculated as MD/(MD + MNM).

Process indicators that reflect the use of evidence-based interventions are presented in Table 5. Two hundred one women gave birth in the hospital, and 96 (48%) received oxytocin as part of active management of third stage of labour (AMTSL). Sixty-six women were diagnosed with severe PPH. Forty-one of these women (62%) delivered in the hospital and 25 women (38%) delivered at home or on their way to the hospital. Twenty-eight women (42%) who were diagnosed with severe PPH had received oxytocin routinely after delivery. The number of women that received oxytocin as treatment was higher: 38 (58%). Ten women received ergometrine and/or misoprostol in addition to oxytocin. Fifty-four women (82%) received intravenous volume replacement, whereas 61 women (92%) received blood transfusion. In four women (6%) hysterectomy was performed.

**Table 5:** Process indicators among women with severe maternal morbidity and mortality

**1. Prevention of PPH**	**n (%)**
Target population: women who delivered in hospital	201 (100%)
Oxytocin use for AMTSL	96 (48%)
Other uterotonic use for AMTSL	2 (1%)
All uterotonic use for AMTSL	96 (48%)
**2. Treatment of PPH**	
Target population: women with PPH	66 (100%)
Delivery in hospital	41 (62%)
Delivery out hospital	25 (38%)
Oxytocin use (routine)	28 (42%)
Other uterotonic use (routine)	-
All uterotonic use (routine)	28 (42%)
Oxytocin use (treatment)	38 (58%)
Other uterotonic use (treatment)	10 (15%)
All uterotonic use (treatment)	39 (59%)
IV-infusion	54 (82%)
Blood products	61 (92%)
Hysterectomy	4 (6%)
CFR	9%
**3. Treatment of eclampsia**	
Target population: women with eclampsia	15 (100%)
Magnesium sulphate use	13 (87%)
Other anticonvulsant use	10 (67%)
Any anticonvulsant use	14 (93%)
CFR	7%
**4. Prevention of caesarean section related infection**	
Target population: women with caesarean section	74 (100%)
Prophylactic antibiotics	49 (66%)
**5. Treatment of sepsis**	
Target population: women with sepsis	30 (100%)
Parenteral therapeutic antibiotics	28 (93%)
CFR	27%
**6. Uterine rupture**	
Target population: women with uterine rupture	21 (100%)
Occurred in hospital	13 (62%)
Occurred out hospital	8 (38%)
Laparotomy	21 (100%)
CFR	5%

*PPH:* postpartum haemorrhage, this is defined as the loss of 500 ml of blood or more; *AMTSL:* active management of third stage of labour; *CFR:* case fatality rate, calculated as MD/(MD + MNM).

Fifteen women were diagnosed with eclampsia, of which 13 women (87%) received magnesium sulphate. Ten women received diazepam, of which nine also received magnesium sulphate, indicating that 93% women received anticonvulsants.

Seventy-four women underwent a caesarean section, of which 49 (66%) received prophylactic antibiotics. Twelve women (16%) developed sepsis after caesarean section and one woman died. Six of the women (50%) who developed sepsis received prophylactic antibiotics.

Twenty-eight of the women (93%) that were diagnosed with sepsis received parenteral therapeutic antibiotics.

The last process indicator regarding uterine rupture revealed that the majority of the ruptures occurred in-hospital (62%). All women underwent laparotomy, and one woman died. CFR for uterine rupture was 5%.

Of all women with life-threatening conditions, 227 women (92%) had physical examination on arrival. One hundred seventy-two women were ill on arrival and 133 (77%) had blood pressure measured, 124 (72%) had pulse rate taken and of 126 women (73%) temperature was noted. Saturation was measured in only 10 women (6%). Full blood count was taken in 155 (90%) of the women that were ill on arrival.

## Discussion

This article presents the results of a prospective cross-sectional study describing the quality of obstetric care in a rural referral hospital in Tanzania using the modified WHO near-miss criteria. It is one of the few studies describing maternal morbidity and mortality in Tanzania [24-28].

MMR in this hospital was 350 maternal deaths per 100,000 live births (95% CI 243–488). Country level MMR is around 460 per 100,000 live births [3,12]. Previous studies in the area revealed MMRs of 362 (95% CI 269–456) and 444 (95% CI 371–517) per 100,000 live births in 1995 and 1996 for household and antenatal clinic surveys, respectively [25]. It is difficult to compare the hospital-based calculated MMR with a population-based MMR because not all women deliver nor die inside the hospital. Most likely hospital-based MMR is an overestimation because 48% of the Tanzanian women deliver at home and 85% of all maternal deaths are identified in-hospital [12,29]. Having this in mind, our estimation of MMR is relatively low compared to the national MMR, as is underlined by previous research from the area. This may be explained by the free service of the hospital for delivery care and the well-established transport services. Furthermore, although the hospital did not have medical specialists, there were frequent long-term expatriate specialists and visiting specialists from nearby referral hospitals.

A recently published systematic review was conducted that included all studies on maternal near miss from 2004 until 2010 [7]. It revealed that inclusion criteria used to identify maternal near miss varied widely and therefore could not easily be compared. The findings showed MNM prevalence rates that varied from 0.04% to 14.98% depending on the different inclusion criteria that were used. These results were further sub-divided into regions and the findings from 14 studies from Africa showed that there is a near-miss prevalence rate ranging from 0.05% to 14.98%. The near-miss prevalence in HLH of 2.48% lies within the lower range of the studies from Africa that were included in this analysis. From the fourteen studies that were analyzed in the sub-group Africa, seven studies used management specific criteria (emergency hysterectomy or ICU admission) and had relatively low near-miss rates, as they did not include all near misses that were admitted to the hospital.

Although maternal mortality and morbidity ratios are relatively low compared to the rest of the country, quality of care can still be improved as is shown by the process indicators. The improvement lies in the up scaling of evidence-based interventions. As suggested by Campbell et al. “we should get on with what works” in order to reduce maternal mortality [28,30]. Implementation of evidence-based interventions such as oxytocin use immediately after delivery for prevention of postpartum haemorrhage can be improved in HLH. Only 48% of the women received routine oxytocin immediately after delivery, while WHO recommends this for all women who give birth [31]. WHO also recommends the use of oxytocin for the treatment of PPH [32], however in HLH it was used in 58% of the cases with severe PPH. PPH was the leading cause of maternal morbidity and the second cause for maternal mortality in HLH with a CFR of 9%. Confronting hospital staff with these low numbers of oxytocin use reveals several possible reasons. Not recording the use of oxytocin in the hospital file is the main reason, followed by lack of protocols, shortage of staff, and high staff turnover resulting in less experienced health care workers. Supply of oxytocin to the ward is not considered a constraint. Although there were no official guidelines in HLH available, local policy was to give 10 IU of oxytocin immediately after birth and it was also the first drug of choice for the treatment of PPH. Use of oxytocin for prevention and treatment of PPH should be scaled up to 100% coverage.

Caesarean section seems to be one of the most important risk factors for postpartum maternal infection [33,34]. Prophylactic antibiotics reduces endometritis and decreases wound infections [35]. At HLH it was used only in 66% of all women who underwent caesarean section. Twelve women (16%) developed sepsis after caesarean section and one woman died. Of the women who developed sepsis, six women (50%) received prophylactic antibiotics. Sepsis accounted for 4% of all maternal morbidities in HLH and the number of women with sepsis after caesarean section may be reduced by this simple precaution. Prophylactic use of antibiotics during caesarean section should be scaled up to 100%.

Use of magnesium sulphate in case of severe pre-eclampsia is recommended as it reduces the risk of eclampsia with 50% and might reduce maternal mortality [36,37]. Eclampsia is not very common at HLH regarding the incidence of 1.6% per 1,000 deliveries, but it does account for 6% of all women with life-threatening conditions. Case fatality rate for eclampsia was 7%. Nearly 90% of all eclamptic women received magnesium sulphate. Hypertensive disorders are typically detected during antenatal care (ANC) visits. ANC coverage in Tanzania is relatively good: 96% of the pregnant women visit the ANC once and 43% visit the ANC four times or more. Most women visit the antenatal clinic 2–3 times. In Manyara region 68.2% of the women have blood pressure measured during ANC visits [12]. ANC visits are a good opportunity to detect hypertensive disorders, and blood pressure should be measured on every occasion.

The majority of uterine ruptures occurred in-hospital, which reflects serious substandard care. Obstructed labour was prevalent in 12% of all maternal morbidities and mortalities. Uterine rupture is one of the medical emergencies with high maternal and perinatal mortality as is reflected by the maternal CFR of 5% [38,39]. Other hospital-based studies from low-income countries have reported case fatality rates ranging from 1 to 13% [40]. Audit can be used to reduce the incidence of uterine rupture as is shown in Malawi [41]. Some of the recommendations that were implemented in Malawi after several audit sessions were training of healthcare workers in order to reduce delay in diagnosing obstructed labour and delay in treatment and implementation of local guidelines for augmentation of labour. Increased supervision should lead to improved documentation and labour management.

There were several limitations to this study. First, it is a single-centre and hospital-based study and therefore findings may not reflect the situation in other settings or the population in general. Second, information is gathered from hospital files, therefore data quality depends on the quality of record keeping. We have tried to remedy this by means of conducting prospective data-collection. Third, for the collection of maternal near miss cases we have used modified WHO near miss criteria, as the original WHO near miss criteria were not applicable in the local context. This has to be taken into account when interpreting the results from this study, as there have been more near miss cases collected with the modified criteria, then would have been with the original criteria [21].

## Conclusion

The present study shows that, although the hospital-based maternal mortality ratio is quite low compared to the country-level maternal mortality ratio, maternal morbidity and mortality remain challenging problems in a rural referral hospital in Tanzania. The WHO near miss criteria used for identification of near miss cases could not all be applied in this low-resource setting. Therefore the inclusion criteria were modified for use in Haydom. Results show that there is a gap between actual and optimal use of evidence-based interventions in women experiencing severe maternal morbidity and mortality. Improvement in the quality of obstetric care can be made through up scaling the use of evidence-based interventions.

## Abbreviations

AMTSL: Active management of third stage of labour; CFR: Case fatality rate; CI: Confidence interval; HLH: Haydom lutheran hospital; ICU: Intensive care unit; MD: Maternal death; MDG: Millennium development goal; MMR: Maternal mortality ratio; MNM: Maternal near miss; PPH: Postpartum haemorrhage; SD: Standard deviation; WLTC: Women with life-threatening conditions.

## Competing interests

EN received a stipend from the Laerdal Foundation for Acute Medicine, Stavanger, Norway. The sponsor of the study had no role in study design, data collection, data analysis, data interpretation, or writing of the report.

## Authors’ contributions

EN was involved in the conception and design of the study, carried out the analysis and wrote the first draft of the paper. EM, HLE, BEO, JVR and JS assisted with the conception and design of the study and contributed to the writing of the paper. BEO, JS and JVR also participated in the classification of diagnoses for all MNM and MD cases. All authors read and approved the final draft of the paper.

## Pre-publication history

The pre-publication history for this paper can be accessed here:

http://www.biomedcentral.com/1471-2393/13/141/prepub

## References

[B1] Hogan MC Foreman KJ Naghavi M Ahn SY Wang M Makela SM Lopez AD Lozano R Murray CJ Maternal mortality for 181 countries, 1980–2008: a systematic analysis of progress towards Millennium Development Goal 5 Lancet 2010 375 1609 1623 10.1016/S0140-6736(10)60518-1 20382417

[B2] Lozano R Wang H Foreman KJ Rajaratnam JK Naghavi M Marcus JR Dwyer-Lindgren L Lofgren KT Phillips D Atkinson C Progress towards Millennium Development Goals 4 and 5 on maternal and child mortality: an updated systematic analysis Lancet 2011 378 1139 1165 10.1016/S0140-6736(11)61337-8 21937100

[B3] World Health Organization, UNICEF, UNFPA, The World Bank Trends in Maternal Mortality: 1990 to 2010. WHO, UNICEF, UNFPA and The World Bank estimates 2012 World Health Organization

[B4] United Nations General Assembly United Nations Millennium Declaration A/RES/55/2. 192000. UN General Assembly, 55th session, agenda item 60(b)

[B5] Pattinson R Near misses: a useful adjunct to maternal death enquiries Br Med Bull 2003 67 231 243 10.1093/bmb/ldg007 14711767

[B6] Say L Pattinson RC Gülmezoglu AM WHO systematic review of maternal morbidity and mortality: the prevalence of severe acute maternal morbidity (near miss) Reprod Heal 2004 1 3 10.1186/1742-4755-1-3 PMC516581 15357863

[B7] Tuncalp O Hindin MJ Souza JP Chou D Say L The prevalence of maternal near miss: a systematic review BJOG 2012 119 653 661 10.1111/j.1471-0528.2012.03294.x 22489760

[B8] Kaye DK Kakaire O Osinde MO Systematic review of the magnitude and case fatality ratio for severe maternal morbidity in sub-Saharan Africa between 1995 and 2010 BMC Pregnancy Childbirth 2011 11 65 10.1186/1471-2393-11-65 21955698 PMC3203082

[B9] Pattinson RC Buchmann E Mantel G Schoon M Rees H Can enquiries into severe acute maternal morbidity act as a surrogate for maternal death enquiries? BJOG 2003 110 889 893 10.1111/j.1471-0528.2003.03044.x 14550357

[B10] Say L Souza JP Pattinson RC Maternal near miss–towards a standard tool for monitoring quality of maternal health care Best Pract Res Clin Obstet Gynaecol 2009 23 287 296 10.1016/j.bpobgyn.2009.01.007 19303368

[B11] World Health Organization Evaluating the quality of care for severe pregnancy complications. The WHO near-miss approach for maternal health 2011 World Health Organization

[B12] National Bureau of Statistics (NBS) [Tanzania] and ICF Macro Tanzania Demographic and Health Survey 2010 Final Report 2011 Dar es Salaam, Tanzania: NBS and ICF Macro

[B13] Koblinsky M Matthews Z Hussein J Mavalankar D Mridha MK Anwar I Achadi E Adjei S Padmanabhan P Marchal B Going to scale with professional skilled care Lancet 2006 368 1377 1386 10.1016/S0140-6736(06)69382-3 17046470

[B14] Graham WJ Criterion-based clinical audit in obstetrics: bridging the quality gap? Best Pract Res Clin Obstet Gynaecol 2009 23 375 388 10.1016/j.bpobgyn.2009.01.017 19299203

[B15] Freedman LP Graham WJ Brazier E Smith JM Ensor T Fauveau V Themmen E Currie S Agarwal K Practical lessons from global safe motherhood initiatives: time for a new focus on implementation Lancet 2007 370 1383 1391 10.1016/S0140-6736(07)61581-5 17933654

[B16] Kruk ME Rockers PC Mbaruku G Paczkowski MM Galea S Community and health system factors associated with facility delivery in rural Tanzania: a multilevel analysis Health Policy 2010 97 209 216 10.1016/j.healthpol.2010.05.002 20537423

[B17] Gabrysch S Campbell OM Still too far to walk: literature review of the determinants of delivery service use BMC Pregnancy Childbirth 2009 9 34 10.1186/1471-2393-9-34 19671156 PMC2744662

[B18] Mathai M To ensure maternal mortality is reduced, quality of care needs to be monitored and improved alongside increasing skilled delivery coverage rates BJOG 2011 118 Suppl 2 12 14 21951495 10.1111/j.1471-0528.2011.03104.x

[B19] Janakiraman V Ecker J Quality in obstetric care: measuring what matters Obstet Gynecol 2010 116 728 732 10.1097/AOG.0b013e3181ea4d4f 20733459

[B20] The United Republic of Tanzania Central Census Office NBoS, President’s Offices, Planning and Privatisation 2002. Population and housing census- village and street statistics, age and sex distribution, Manyara Region 2005 Dar es Salaam

[B21] Nelissen E Mduma E Broerse J Ersdal H Evjen-Olsen B Van Roosmalen J Stekelenburg J Applicability of the WHO Maternal Near Miss Criteria in a Low-Resource Setting PLoS One 2013 8 e61248 10.1371/journal.pone.0061248 23613821 PMC3629023

[B22] World Health Organization International statistical classification of diseases and related health problems 2011 10th revision, edition 2010. World Health Organization 10.1016/j.biopsych.2010.10.023 21195389

[B23] Lauritsen J Bruus M EpiData. A comprehensive tool for validated entry and documentation of data 2003 Odense, Denmark: The EpiData Association

[B24] Nyamtema AS De Jong AB Urassa DP Van Roosmalen J Using audit to enhance quality of maternity care in resource limited countries: lessons learnt from rural Tanzania BMC Pregnancy Childbirth 2011 11 94 10.1186/1471-2393-11-94 22088168 PMC3226647

[B25] Olsen BE Hinderaker SG Kazaura M Lie RT Bergsjo P Gasheka P Kvale G Estimates of maternal mortality by the sisterhood method in rural nothern Tanzania: a household sample and an antenatal clinic sample BJOG 2000 107 1290 1297 10.1111/j.1471-0528.2000.tb11622.x 11028583

[B26] Mbaruku G Van Roosmalen J Kimondo I Bilango F Bergstrom S Perinatal audit using the 3-delays model in western Tanzania Int J Gynaecol Obstet 2009 106 85 88 10.1016/j.ijgo.2009.04.008 19439297

[B27] Francesconi P Pisani V Estimating maternal mortality by the Sisterhood method in Iringa District Tanzania. Trop Doct 2001 31 220 10.1177/004947550103100416 11676061

[B28] Sorensen BL Elsass P Nielsen BB Massawe S Nyakina J Rasch V Substandard emergency obstetric care - a confidential enquiry into maternal deaths at a regional hospital in Tanzania Trop Med Int Health 2010 15 894 900 10.1111/j.1365-3156.2010.02554.x 20545917

[B29] Olsen BE Hinderaker SG Lie RT Bergsjo P Gasheka P Kvale G Maternal mortality in northern rural Tanzania: assessing the completeness of various information sources Acta Obstet Gynecol Scand 2002 81 301 307 10.1034/j.1600-0412.2002.810404.x 11952458

[B30] Campbell OM Graham WJ Strategies for reducing maternal mortality: getting on with what works Lancet 2006 368 1284 1299 10.1016/S0140-6736(06)69381-1 17027735

[B31] World Health Organization WHO recommendations for the prevention of postpartum haemmorhage 2007 World Health Organization

[B32] World Health Organization WHO guidelines for the management of postpartum haemorrhage and retained placenta 2009 World Health Organization 23844453

[B33] Gibbs RS Clinical risk factors for puerperal infection Obstet Gynecol 1980 55 178S 184S 10.1097/00006250-198003001-00045 6990333

[B34] Declercq E Barger M Cabral HJ Evans SR Kotelchuck M Simon C Weiss J Heffner LJ Maternal outcomes associated with planned primary cesarean births compared with planned vaginal births Obstet Gynecol 2007 109 669 677 10.1097/01.AOG.0000255668.20639.40 17329519

[B35] Smaill FM Gyte GM Antibiotic prophylaxis versus no prophylaxis for preventing infection after cesarean section Cochrane Database Syst Rev 2010 CD007482 10.1002/14651858.CD007482.pub2 PMC4007637 20091635

[B36] Duley L Gulmezoglu AM Henderson-Smart DJ Chou D Magnesium sulphate and other anticonvulsants for women with pre-eclampsia Cochrane Database Syst Rev 2010 CD000025 10.1002/14651858.CD000025.pub2 PMC7061250 21069663

[B37] Altman D Carroli G Duley L Farrell B Moodley J Neilson J Smith D Do women with pre-eclampsia, and their babies, benefit from magnesium sulphate? The Magpie Trial: a randomised placebo-controlled trial Lancet 2002 359 1877 1890 12057549 10.1016/s0140-6736(02)08778-0

[B38] Ali AA Adam I Maternal and perinatal outcomes of uterine rupture in the Kassala Hospital, east Sudan: 2006–2009 J Obstet Gynaecol 2011 31 48 49 10.3109/01443615.2010.522268 21280993

[B39] Aboyeji AP Ijaiya MD Yahaya UR Ruptured uterus: a study of 100 consecutive cases in Ilorin, Nigeria J Obstet Gynaecol Res 2001 27 341 348 10.1111/j.1447-0756.2001.tb01283.x 11794821

[B40] Hofmeyr GJ Obstructed labor: using better technologies to reduce mortality Int J Gynaecol Obstet 2004 85 Suppl 1 S62 72 15147855 10.1016/j.ijgo.2004.01.011

[B41] van den Akker T Mwagomba B Irlam J Van Roosmalen J Using audits to reduce the incidence of uterine rupture in a Malawian district hospital Int J Gynaecol Obstet 2009 107 289 294 10.1016/j.ijgo.2009.09.005 19846089

